# Mace Poisoning: Accidental Toxic Ingestion in a Child Leading to a Reversible Coma

**DOI:** 10.7759/cureus.76284

**Published:** 2024-12-23

**Authors:** Ayesha Imtiaz, Bassam M Almutairy, Taghreed M Almutairi, Moatasem M Aloufi, Ahmed H Almutairi

**Affiliations:** 1 Pediatric Emergency Department, King Saud Medical City, Riyadh, SAU; 2 Pediatric Emergency Department, King Fahad Medical City, Riyadh, SAU; 3 Psychology Department, Ministry of Defence, Riyadh, SAU

**Keywords:** accidental toxic ingestion, child, hallucinogen, mace poisoning, pediatric

## Abstract

Nutmeg and mace are commonly known for their medicinal and culinary properties. The chemical compounds found in nutmeg and mace, notably myristicin, elemicin, and safrole, have been implicated in the psychoactive and anticholinergic effects that are the result of acute toxicity. Cases of mace toxicity are not as commonly reported as nutmeg toxicity. We report a six-year-old child, who presented with serotonergic and anticholinergic symptoms, with an altered level of consciousness and respiratory acidosis after unintentionally ingesting six pieces of mace. She recovered with supportive care alone and was discharged 36 hours post-ingestion. Myristicin acts by moderately inhibiting monoamine oxidase, causing anticholinergic symptoms. It also leads to the formation of the metabolite 3-methoxy-4,5-methylendioxy amphetamine (MMDA), responsible for its psychedelic effects. Both intentional and unintentional toxicity by nutmeg have been reported widely, the latter occurring more commonly in children less than 13 years of age. This case highlights the potential toxicity of mace ingestion and demonstrates the need for heightened awareness among families to prevent accidental exposures.

## Introduction

Nutmeg, a seed from the plant *Myristica fragrans*, and mace, its dried lacy aril, have long been valued globally for their medicinal and culinary uses [1]. The chemical composition of nutmeg and mace consists of a myristic acid and a mixture of derivatives of terpenes and alkenyl benzene. The aromatic properties of the seed are due to the presence of monoterpenes and other aromatic compounds. 80% of the alkenyl benzene derivatives are in the form of myristicin, safrole, and elemicin. These are biologically active compounds, present in different compositions, but common to both nutmeg and mace, and are responsible for their anti-inflammatory, anti-convulsant, and anti-microbial properties [1-3]. Of these chemical compounds, myristicin and elemicin have anticholinergic and psychotropic properties and are shown to be associated with toxic effects on the central nervous system (CNS) in animal studies [4].

Acute toxicity can result from its recreational use - more common in adolescents - or from accidental ingestions [5,6]. Symptoms commonly occur one to eight hours after a toxic ingestion and can last up to 36 hours [7]. It can affect the gastrointestinal (GI) system, causing nausea, vomiting, abdominal pain, or constipation [8]. Cardiovascular (CV) effects are usually the result of a weak monoamine oxidase inhibitor effect causing tachycardia, hypertension, or hypotension. CNS toxicity commonly results in anticholinergic effects and psychotropic effects causing anxiety, confusion, hallucinations, psychosis, and seizures with altered levels of consciousness ranging from delirium to coma [8]. Nutmeg poisoning alone is not commonly known to be fatal, with only two reported fatal cases: one more than a century ago and another a few decades ago [9,10].

Cases of acute toxicity from nutmeg have been widely reported by poison control centers in the Americas and European countries in the past decades, though poisoning by mace alone has not been reported often [5,6,10]. To the best of our knowledge, this is the first reported case of mace toxicity in the Kingdom of Saudi Arabia (KSA).

## Case presentation

**Figure 1:**
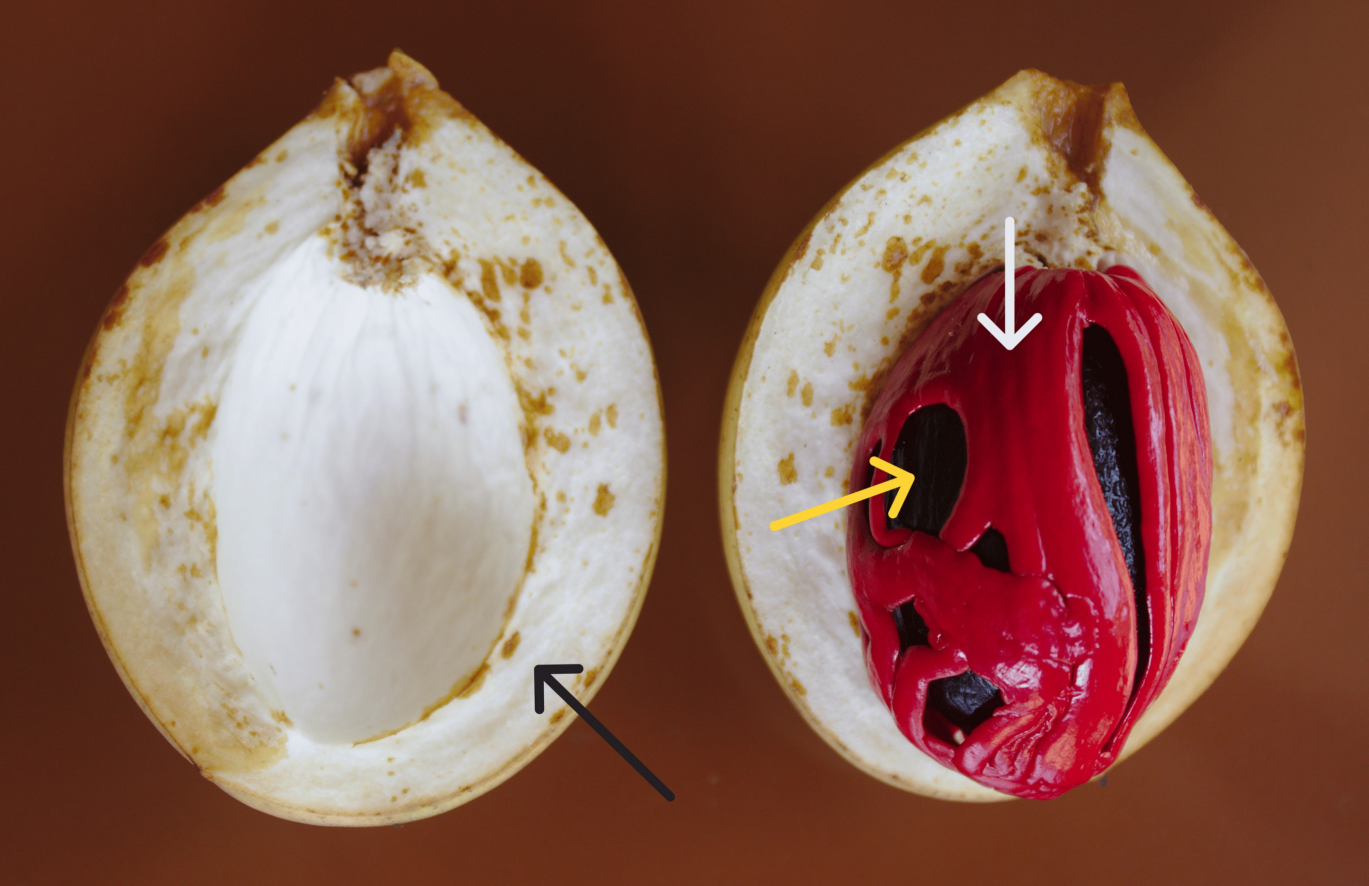
A nutmeg fruit (black arrow) split open to show the seed (yellow arrow) with the surrounding aril (white arrow), also known as mace.

We present the case of a six-year-old girl, previously healthy with no known medical or surgical conditions, who was brought to the Emergency Department (ED) by her parents, who reported that she was unusually drowsy and difficult to arouse from sleep, beyond her usual wake-up time. They also reported that she had gone to bed early the night before and was unusually drowsy before falling asleep. They denied any history of head trauma, fever, vomiting, headache, or diarrhea. The only medications reported to be present at home were acetaminophen tablets, and the family denied any possibility of the child having ingested any of these medications due to good supervision. The only concern expressed by the family was that the grandmother had brought fresh nutmeg with its lacy red covering (mace) and had placed six pieces out in the sun to dry (Figure 1) [11]. Around 6 PM, the patient's older sibling informed the parents that the patient had peeled the mace off all the drying nutmeg and ingested it.

At presentation, the child’s heart rate was 127 per minute, temperature was 37° Celcius, respiratory rate was 24 per minute, and blood pressure was 95/63 mmHg. Her Glasgow Coma Scale (GCS) was 8/15 in triage, with an eye opening to painful stimuli, no verbal response, and purposeful motor response to pain. After the initial vitals were obtained, her heart rate and respiratory rate came down to and remained between the ranges of 81 and 92 beats per minute and 15 and 20 breaths per minute, respectively. Her airway, breathing, and circulation were stable. Further neurological examination demonstrated pupils that were 4 mm, symmetrical, and reactive to light bilaterally. She had fasciculations of the muscles of the upper and lower limbs with hypertonia and cogwheel rigidity on examination. Deep tendon reflexes were normal, including the babinski reflex, which was negative. There was no neck rigidity and no meningeal signs.

Investigations

A 12-lead ECG showed a sinus rhythm. A CT brain scan was done to rule out any acute intracranial pathology, which was normal. Blood glucose at presentation was 111 mg/dL. Initial venous blood gas (VBG) showed respiratory acidosis with a pH of 7.28 and pCO_2_ of 53 mmHg. Base deficit was 2.3 mmol/L and HCO_3_ was 24.9 mmol/L. No electrolyte abnormalities were detected, and liver and renal function tests were normal. The toxicology center was consulted, which advised pediatric intensive care unit (PICU) admission and supportive management. Pediatric neurology and PICU were consulted for their opinion on the possibility of encephalitis and for supportive management, respectively.

Outcome

During her three-and-a-half-hour stay in the ED, she was given supportive management by keeping her nil per os (NPO), administering intravenous fluids, and was under CV monitoring. Near the end of her ED stay, her GCS improved to 14/15, with spontaneous eye opening, oriented verbal responses, and purposeful movement to pain (E4 V5 M5). Her tone normalized, and vital signs continued to be within the acceptable normal range for her age. A repeated VBG showed a pCO_2_ of 47 mmHg, pH of 7.29, HCO_3_ of 22.6 mmol/L, and a base deficit of 4 mmol/L, indicating improving respiratory acidosis. Eighteen hours post-ingestion and less than four hours after arrival at the pediatric ED, she was admitted to the observation unit. Neurological evaluation 20 hours post-ingestion and six hours post-presentation revealed normal findings. She was discharged from the ward the next day with an uneventful admission.

## Discussion

Nutmeg is a commonly abused household spice, along with vanilla, and its abuse has been widely reported [12]. Mace has similar chemical constituents to nutmeg, but in different compositions; however, mace toxicity has not been reported as frequently. Myristicin, the main psychoactive compound in nutmeg and mace, is an alkyl-benzene derivative along with elemicin and safrole. Myristicin causes its toxic effects by two mechanisms. It primarily acts to inhibit monoamine oxidase, the enzyme responsible for the breakdown of serotonin, nor-adrenaline, and dopamine, leading to symptoms of serotonin toxicity [13]. The psychedelic effect of myristicin is attributed to its metabolite 3-methoxy-4,5-methylendioxy amphetamine (MMDA). Elemicin, which is structurally similar to the psychedelic alkaloid mescaline, causes hallucinations and has anticholinergic effects as well [13].

A 10-year analysis of nutmeg exposure from the Illinois Poison Center showed that 47% of exposures to nutmeg were intentional (for recreational use due to their hallucinogenic properties) [6]. The incidence of intentional exposures was double in the adolescent age group compared to those above 19 years of age, highlighting the risk in children of this age group. All children under the age of 13 years had unintentionally been exposed to nutmeg toxicity [6]. The most common symptoms reported from this cohort of patients were CNS (hallucinations, agitation, drowsiness, and ocular dysfunction), CV (tachycardia), and GI symptoms (nausea and vomiting). Similar results were demonstrated by Texas poison centers over six years, where none of the intentional exposures were recorded in children below 13 years, and the majority of these recreational exposures occurred in adolescents [5]. These reports are similar to our case where the child was six years old, the exposure was accidental, and the most obvious symptoms were neurological and CV.

Anticholinergic effects from nutmeg toxicity were reported in an 18-year-old who ingested about 50 gm of grated nutmeg for recreational use [8]. She reported to have been agitated and anxious, with complaints of dry mouth, thirst, nausea, and dizziness. Similar to the timeline in our case, her symptoms completely resolved 16 hours after ingestion. Electrolyte abnormalities have not commonly been reported with myristicin poisoning. The only case reported with electrolyte abnormalities of mild hyponatremia and hypokalemia occurred accidentally in a 33-year-old female, who ingested 5-10 gm of nutmeg in her food [14]. She drank more than 1 L of water within a short period of time when she subsequently became symptomatic with dry mouth. At a presentation to the ED, she had an obvious tremor and dilated, unequal pupils. The physical exam and lab findings were attributed to the nutmeg ingestion that had occurred. This was contrary to our case, except that our patient was in an obtunded state, with a depressed level of consciousness, and had exhibited respiratory acidosis.

Nutmeg ingestion is not known to be fatal. Of the two reported cases of fatal ingestion, one involved an 8-year-old boy (reported in 1908) who ingested two nutmeg seeds, entered a state of stupor, and died 24 hours later [9]. This can be attributed to the lack of advanced technology, respiratory support, and monitoring that became available in later decades. The more recent fatal case involved a 55-year-old woman who had co-ingested flunitrazepam, a benzodiazepine [10]. The cause of her death could have been attributed to either one of the ingestions.

Mace and nutmeg are commonly available household spices, and most of the accidental as well as non-accidental ingestions have been shown to occur in children [6]. Therefore, the importance of increasing parental awareness about the toxic effects of mace and nutmeg cannot be emphasized enough. This can be accomplished by carrying out targeted awareness campaigns in the community that highlight the potential toxicity of these substances [15].

## Conclusions

Nutmeg ingestions, both intentional and unintentional, have been reported globally across centuries, but toxicity from mace ingestion is less well-known and reported. This case underscores the need for increased awareness about the toxic effects of mace, in order to improve patient education and develop measures to prevent toxic pediatric ingestion.
